# Bone age evaluation in an ethnically diverse cohort of children with premature adrenarche

**DOI:** 10.1038/s41390-025-04459-2

**Published:** 2025-10-03

**Authors:** Liya Kerem, Marwa Tuffaha, Aluma Chovel Sella, Luz Elena Castellanos, Jonanlis Ramirez Alcantara, Takara Stanley

**Affiliations:** 1 Division of Pediatric Endocrinology, Department of Pediatrics, Hadassah Hebrew University Medical Center, The Hebrew University of Jerusalem, Jerusalem, Israel; 2 Division of Pediatric Endocrinology, Boston Children’s Hospital, Harvard Medical School, Boston, MA USA; 3 The Jesse Z. and Sara Lea Shafer Institute of Endocrinology and Diabetes, National Center for Childhood Diabetes, Schneider Children’s Medical Center of Israel, Petah Tikva, Israel; 4 Department of Endocrine Neoplasia and Hormonal Disorders, The University of Texas, Anderson Cancer Center, Houston, TX USA; 5 Division of Pediatric Endocrinology, Department of Pediatrics, Nicklaus Children’s hospital, Miami, FL USA; 6 Division of Pediatric Endocrinology, Division of General Academic Pediatrics, Massachusetts General Hospital for Children, Boston, MA USA

## Abstract

**Background:**

Bone age (BA) assessment is an essential tool in pediatric endocrinology, used to assess growth and perturbations in pubertal onset. BA advancement is common in children with premature adrenarche (PA), potentially leading to additional evaluation or intervention. The extent to which BA advancement reflects variation in metabolic and demographic factors, including body mass index (BMI), sex, race, and ethnicity, remains insufficiently characterized.

**Methods:**

We conducted a retrospective chart review of 296 children (72% female, mean age 7.3 ± 1.6 years) with isolated PA seen at a tertiary pediatric endocrinology clinic. Absolute and standardized BA advancement were analyzed in relation to BMI, sex, race, and ethnicity. Multivariate linear regression adjusted for age and covariates.

**Results:**

BA advancement was greater in children with obesity (19.2 ± 15.1 months) versus those below the 95th% (11.4 ± 13.5), and in males (19.9 ± 14.3) versus females (12.4 ± 14.3). White race was associated with lower advancement (*p* = 0.02). BMI (*p* < 0.0001), male sex (*p* < 0.0001), and Hispanic vs. White ethnicity (*p* = 0.023) significantly affected standardized BA advancement.

**Conclusion:**

BMI, sex, and race/ethnicity influence BA advancement in PA, supporting individualized interpretation and further study of clinical implications.

**Impact:**

- Bone age (BA) advancement is an important consideration in the diagnostic workup of children with premature adrenarche. In this diverse cohort, BMI status, sex, race, and ethnicity were significantly associated with BA advancement, suggesting that both metabolic and demographic factors influence skeletal maturation. While BA advancement in obesity and premature adrenarche is recognized, this study underscores their combined effects and the variability across populations. These findings point to limitations in current BA standards and support the need for individualized interpretation. Further research should explore how BA advancement in obesity and across ethnic groups affects adult height and long-term outcomes.

## Introduction

The evaluation of skeletal maturation and determination of bone age (BA) in relation to the chronological age is one of the main tools used by endocrinologists to assess the presence of early puberty. Linear growth, which is achieved by the elongation of long bones at the growth plate through endochondral ossification, is tightly regulated by an intricate network of endocrine signals, locally produced growth factors, and paracrine factors.^1^ During puberty and sexual maturation, epiphyseal fusion, which reflects the resorption of the growth plate, results in deceleration and eventually termination of linear growth. Estrogen plays a critical role in this process by accelerating the growth plate senescence program, thereby promoting earlier epiphyseal fusion.^2^ Consequently, the finding of significantly advanced bone age in a child presenting with early puberty results in further, usually comprehensive workup for precocious gonadal puberty and pathological causes of androgen secretion, and it may increase the likelihood of initiating pharmacological interventions to halt puberty and avoid compromised final adult height.^3^

Whereas precocious gonadal puberty is reflected clinically by breast development in girls and testicular enlargement in boys, premature adrenarche (PA) is defined as the appearance of pubic and/or axillary hair, potentially accompanied by sebaceous and apocrine gland development before 8 years in girls and 9 years in boys. It is accompanied by a peripheral increase in adrenal androgens (mainly dehydroepiandrosterone sulfate; DHEAS) released from the zona reticularis of the adrenal cortex to a level consistent with the patient’s Tanner stage for pubic hair. Importantly, idiopathic PA is diagnosed in the absence of precocious gonadal puberty and when other pathologies have been ruled out (e.g., adrenal or gonadal virilizing malignancies and congenital enzymatic defects of steroidogenesis).^4^ PA is associated with obesity in some,^5^ but not all studies^6^ and both PA and childhood obesity are independently associated with bone age advancement.^7^ Therefore, in individuals with both obesity and PA, the use of bone age to determine the need for further laboratory evaluation and the use of bone age to predict adult predicted height are both potentially hampered. Our understanding of the effects of BMI as well as demographic variables on bone age advancement is incomplete, yet it is essential to the appropriate use of bone age as a tool to guide further clinical evaluation.

To date, it is undetermined whether advanced BA in the setting of isolated PA results in compromised achieved adult height relative to the patient’s genetic potential, estimated by the mid-parental height (MPH). While some studies found that BA advancement has a negligible effect on adult height prediction^8^ or achieved height,^6,9,10^, their cohorts of patients with PA included mostly or exclusively girls, and body mass index (BMI) status, sex, race, or ethnicity were not included in the analyses as explanatory variables.

The goal of our retrospective study was to examine the effect of BMI status on BA advancement in a diverse cohort of both boys and girls from different ethnicities with isolated PA.

## Methods

### Study design

We identified through the Institutional Research Patient Data Registry all patients between the ages of 4–18 years who had bone age X-ray films done at the Massachusetts General Hospital (MGH) between January 2008 through December 2019. The BA was determined by a radiologist based on the Greulich and Pyle atlas.^11^ We obtained Institutional Review Board approval prior to chart review, and the requirement for informed consent was waived. Inclusion criteria for the study required the presence of the following information in the medical record: (1) ICD-9 or ICD-10-CM codes that could reflect the diagnosis of premature adrenarche (i.e., ICD-9: 259.1; ICD-10: E30.1; E30.8 E30.9 and E27.0), (2) height and weight measurements recorded within one month prior to or following BA testing and (3) an in-person clinical evaluation by a pediatric endocrinologist confirming the diagnosis of isolated PA. Exclusion criteria were applied based on both chart review and ICD-9/ICD-10 coded diagnoses. Diagnoses coded as “precocious puberty” (ICD-9: 259.1; ICD-10: E30.1; E30.8 and E30.9) were further reviewed to identify cases of central precocious puberty and isolated thelarche, which were both excluded. Additional exclusions included congenital adrenogenital disorders (ICD-9: 255.2; ICD-10:E25.0) and treatment with GnRH analogs to halt puberty. In addition to excluding patients with endocrine diagnoses other than PA that could clearly explain their presentation, we also excluded cases in which diagnoses potentially affecting growth and development were coded more than once during the study period. These exclusions were based solely on ICD codes and were not followed by individual chart review. Diagnoses included celiac disease (ICD-9:579.0; ICD-10: K90.0), history of abuse (ICD-9:995.5x; ICD-10:T74.x, T76.x), chronic steroid treatment (ICD-9:V58.65; ICD-10:Z79.52), and skeletal dysplasia (ICD-9: 756.4; ICD-10:Q77.9). Based on these criteria, all patients in the cohort had isolated PA confirmed by a pediatric endocrinologist, and none were diagnosed with an underlying cause of PA such as congenital adrenal hyperplasia. Although all cases were clinically assessed and deemed consistent with PA, the absence of laboratory evaluation in some instances limits our ability to confirm a definitive diagnosis of idiopathic PA across the entire cohort.

### Statistical analysis

To examine the association between BA advancement and the baseline characteristics of children presenting with premature adrenarche, we defined BA advancement as the standardized difference between BA and chronological age. This was calculated by standardizing the difference according to the known relevant SDs for age and biological sex as specified by the Brush Foundation study of child growth and development.^12^ We analyzed data available from the first BA testing for each patient.

BMI status was established according to the Centers for Disease Control's age-and-sex-specific growth charts.^13^ Healthy weight was defined as BMI greater than the 5th and less than the 85th percentiles, overweight as BMI at or above the 85th and lower than the 95th percentiles, and obesity as BMI at or above the 95th percentile for age and sex.

Adult predicted height (APH) was calculated using the Bayley-Pinneau tables^14^, which provide estimates of skeletal maturation based on bone age. We determined the alignment of each BA assessment relative to chronological age (delayed, consistent, or advanced) according to the Brush foundation tables, and subsequently calculated the adult height with the corresponding Bayley-Pinneau estimates.

We conducted analyses using R 4.2.2. All descriptive statistics for continuous variables are expressed as means ± standard deviation (SD) and percentages for categorical variables unless otherwise specified. The Kolmogorov–Smirnov test was applied to verify the normal distribution of the data. We used Student’s *t*-test to compare continuous variables and the Chi-squared, Fisher’s exact test, and the Wald test to compare categorical variables, as appropriate. One-way ANOVA was applied to determine differences in BA advancement between patients of different races and ethnicities, and two-way ANOVA was used to examine the degree of BA advancement based on sex and BMI status. Finally, multivariate analyses were performed to examine the effect of BMI status on standardized BA advancement, adjusting in stages for sex, age, race, and ethnicity, using linear regressions. A *p*-value < 0.05 was considered statistically significant.

## Results

**Fig. 1:**
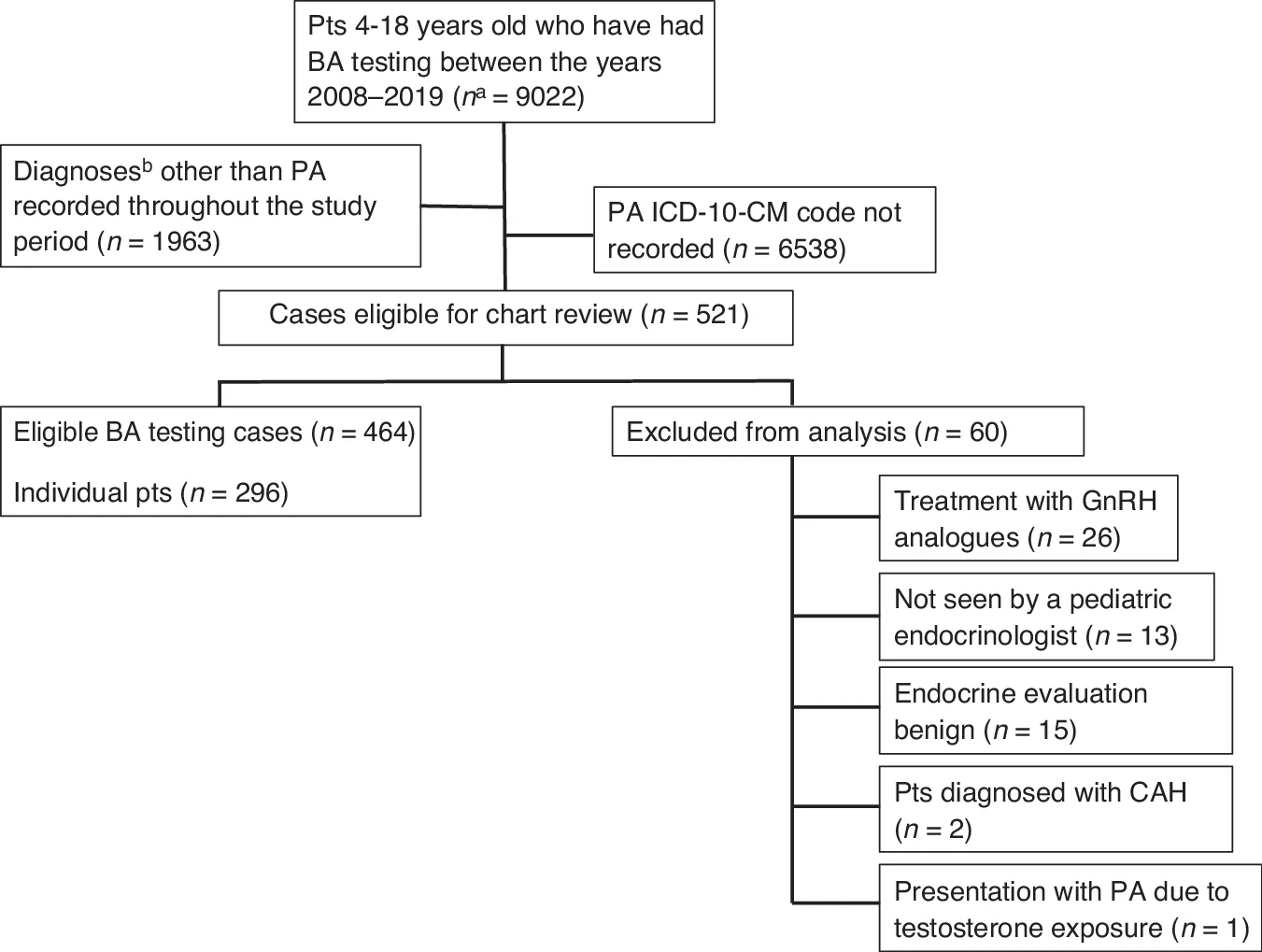
Study flowchart. **a** n refers to the number of BA testing. **b** Patients were excluded if they had the following diagnoses recorded in their charts: Genetic syndrome, endocrine conditions other than premature adrenarche, and pituitary disorders. Cases were also excluded if the following diagnoses were recorded in their charts twice or more throughout the research period: Chronic disease, abuse, chronic steroid use, bone disease, and celiac disease. Pts patients, BA bone age, PA premature adrenarche, BMI body mass index.

Of the 9022 cases of bone age testing found, 1963 were excluded due to diagnoses other than PA (see Fig. 1 for details). Next, we excluded all 6538 cases that did not have an ICD-9 or ICD-10-CM code for PA recorded at least once in their charts throughout the research period. A chart review of all 521 remaining cases was performed to confirm the diagnosis of PA and extract additional required data. Following the chart review, additional cases were excluded from the analysis (see Fig. 1 for details), and the final cohort included 464 eligible cases of BA testing, representing 296 individual patients. Data collected from the chart review included weight and height measurements done within one month of BA testing, a formal BA reading provided by Radiology, and self-reported race and ethnicity of parents. We also recorded the Near Final Height (NFH) available for each participant from the main MGH and affiliated satellite clinics. NFH was defined as the last recorded height at age 14 years or greater for girls and 16 years or greater for boys. Visit summaries were reviewed to confirm the diagnosis of PA, exclude other diagnoses not recorded with an ICD-9 or ICD-10-CM code, and review treatment plans. Overall, 60 patients were excluded from the analysis due to the following reasons (see Fig. 1): Eight patients (26 cases of BA testing) presented clinically and biochemically with PA and did not meet criteria for central precocious puberty; however, they were treated with GnRH agonists due to significant BA advancement and/or increase in height velocity and were therefore excluded from the analysis. Eleven patients (13 cases of BA testing) who were seen by their pediatrician and not evaluated personally by pediatric endocrinology were excluded from the analysis as well. Ten patients (15 cases of BA testing) referred for workup of PA had benign evaluation (either due to their age at presentation not being consistent with premature adrenarche or the lack of clinical findings sufficient to establish the diagnosis). Two patients (2 cases of BA testing) were found to have non-classical congenital adrenal hyperplasia, and one patient was found to have hyperandrogenism induced by testosterone exposure. Children with a BMI lower than the 5^th%^ were omitted from the study due to their rarity (1.3%) and potentially different etiology or underlying diagnosis for premature adrenarche (e.g., Silver-Russell^15^ and Weidemann-Steiner syndromes^16^). As a sensitivity analysis, the main analyses were repeated with these participants, yielding similar results. Of note, while data analysis was performed using the first BA testing of each one of the 296 individual patients, all available visit summaries were reviewed to exclude other diagnoses, to confirm the diagnosis of PA, and to exclude patients in whom pharmacotherapy was initiated to attenuate puberty.

Of the 296 eligible patients for analysis, 72% were females. Mean age at diagnosis was 7.32 (±1.59) years. Almost half (46.5%) of patients were White, 18.6% Hispanic or Latino and 14% Black or African-American. The mean BMI Z score for the entire sample was 1.25 ± 0.96 and the mean difference between BA and chronological age was 14.49 ± 14.65 months (see Table 1 for details).

**Table 1:** Demographic characteristics and clinical features of study population.

Characteristics	Mean (SD) or *N* (%)
***N***	296
**Demographics**	
Age at diagnosis, years	7.32 ± 1.59
Biological sex (*n*, %)	Female 213 (72%) Male 83 (28%)
**Race and Ethnicity (***n***, %)**	
White	138 (46.5%)
Black or African-American	55 (18.6%)
Hispanic or Latino	41 (13.9%)
Asian*	4 (1.4%)
Other	58 (19.6%)
**Anthropometric measurements at diagnosis**	
Mean weight Z score	1.37 ± 1.07
Mean Height Z score	0.87 ± 1.06
Mean BMI (kg/m2 ± SD)	19.63 ± 3.79
Mean BMI Z score	1.25 ± 0.96
Mean BMI percentile	81.31 ± 21.99
**BA-CA (difference in months** ± **SD)**	14.49 ± 14.65
**BA reading (months, years)**	102.38 (8.53 yrs) ± 25.57 (2.13 yrs)

*Due to the small group of Asian patients in this cohort, Asian and “other” (i.e., individuals who elected not to specify their race and ethnicity and patients with multiple racial and ethnical origins) were grouped together for the purpose of additional analyses.

**Fig. 2:**
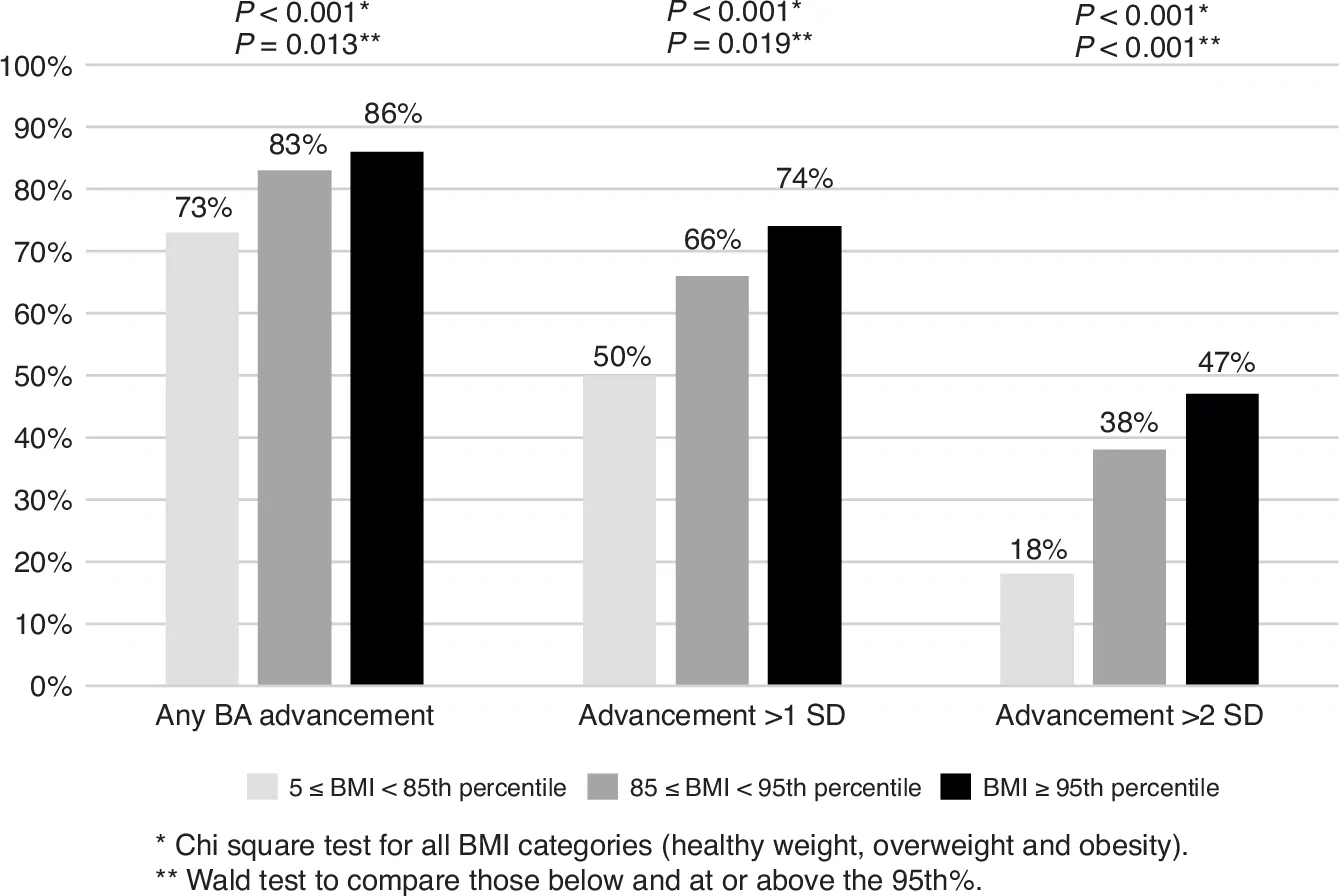
Percentage of patients in each BMI category based on bone age advancement (any bone age advancement, advancement greater than 1 standard deviation and greater than 2 standard deviations). * Chi square test for all BMI categories (healthy weight, overweight and obesity). ** Wald test to compare those below and at or above the 95^th%^.

While the majority of patients demonstrated some degree of BA advancement regardless of their BMI status, significant BA advancement (greater than 2 SDs) was observed in only 18% of those with a healthy weight compared with 47% of those with obesity (*p* < 0.001; see Fig. 2 for details). Mild BA advancement was also more prevalent in those with obesity (74.4%) compared with those with a healthy weight (50%, *p* < 0.001). Both the absolute and the standardized difference between BA and chronological age were significantly different when comparing patients below and above the 95^th%^ (Table 2, absolute difference of 11.42 (±13.52) months in patients with BMI below the 95^th%^ vs. 19.18 (±15.13) months in patients with BMI at or above the 95^th%^; standardized difference of 1.16 (±1.39) SD in patients with BMI below the 95^th%^ vs. 1.96 (±1.56) SD in patients with BMI at or above the 95^th%^ for age and sex, *p* < 0.001 for both comparisons).

**Table 2:** Mean absolute difference (in months) and standardized difference between bone age and chronological age in patients with premature adrenarche, by BMI status.

	BMI < 95^th%^	BMI ≥ 95^th%^	95% CI for the difference	*P*-value
	Mean (SD)	Mean (SD)		
***N***	179	117		
**Absolute difference**	11.42 (±13.52)	19.18 (±15.13)	(4.44, 11.07)	<0.0001
**Standardized difference**	1.16 (±1.39)	1.96 (±1.56)	(0.46, 1.14)	<0.0001

The standardized difference between BA and chronological age did not differ between patients with an initial evaluation obtained at an age younger than 9 years (1.54 ± 1.55) compared to those evaluated at 9 years of age or older (1.47 ± 1.51), *p* = 0.8.

Males with isolated PA demonstrated significantly greater BA advancement [absolute difference 19.94 ± 14.26 months, 95% CI (3.94, 11.21); standardized difference 2.19 ± 1.57, 95% CI (0.62, 1.35)] compared with females [absolute difference 12.36 ± 14.28; standardized difference 1.20 ± 1.39, *p* < 0.001 for both comparisons)].

**Fig. 3:**
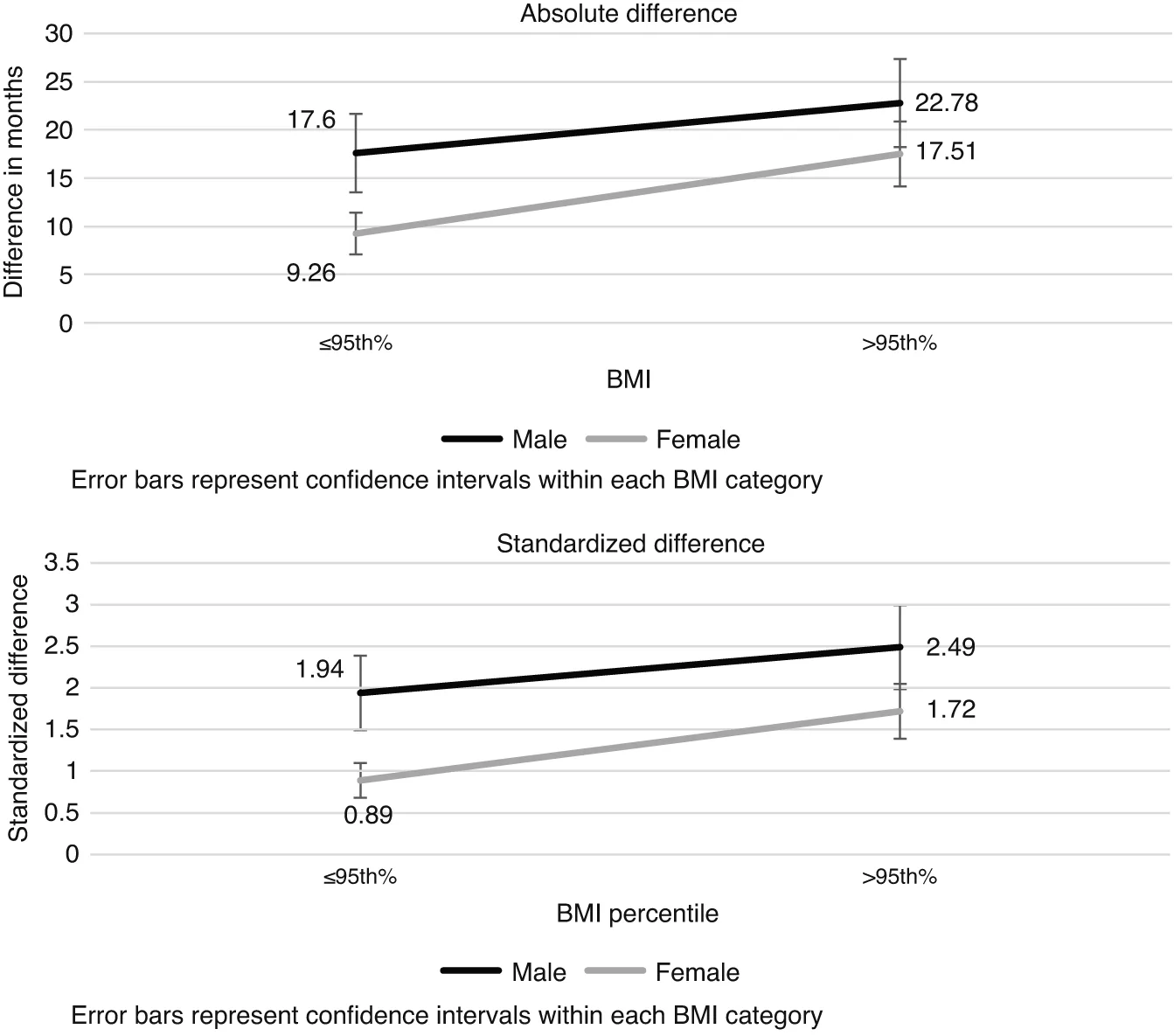
Effects of Obesity status (BMI at or above the 95^th%^ or lower than the 95^th%^) on bone age advancement depending on sex. Error bars represent confidence intervals within each BMI category

Similar significant differences between those with high and normal BMI were found when stratifying for male and female separately (see Table 3 and Fig. 3). No interaction was found between sex and BMI (p value for interaction was 0.391 for the difference parameter and 0.450 for the standardized).

**Table 3:** Mean absolute (in months) and standardized difference between bone age and chronological age in males and females with premature adrenarche, by BMI status.

Sex	BMI percentiles	Difference Mean ± SD	Standardized Mean ± SD
**Female**	**Total (***n* = **213)**	12.36 ± 14.28	1.20 ± 1.39
	**<95**^**th%**^ **(***n* = **133)**	9.26 ± 12.68	0.89 ± 1.22
	≥**95**^**th%**^ **(***n* = **80)**	17.51 ± 15.35	1.72 ± 1.50
**Male**	**Total (***n* = **83)**	19.94 ± 14.26	2.19 ± 1.57
	**<95**^**th%**^ **(***n* = **46)**	17.65 ± 14.08	1.94 ± 1.54
	≥**95**^**th%**^ **(***n* = **37)**	22.78 ± 14.16	2.49 ± 1.564
**P value**	**Sex effect**	<0.001	0.001
	**BMI effect**	0.001	<0.001
	**Interaction**	0.391	0.450

There was a trend towards significantly different BA advancement between all ethnic and race groups (see Table 4 for details), being more pronounced in children with Hispanic origin (absolute difference 18.90 months ±15.29; standardized difference 1.92 ± 1.52) compared with Black or African (absolute difference 16.09 months ±14.61; standardized difference 1.65 ± 1.51) versus White (absolute difference 12.40 months ±14.32; standardized difference 1.26 ± 1.46; *p* = 0.065 for the absolute difference and *p* = 0.067 for the standardized difference). Patients reporting White race exhibited significantly lesser BA advancement compared with other races and ethnic origins (*p* = 0.022 for absolute difference and *p* = 0.02 for standardized difference). We did not find an interaction between BMI status and race and ethnicity with regard to BA advancement (Table 4).

**Table 4:** Mean absolute (in months) and standardized difference between bone age and chronological age in patients with premature adrenarche based on race and ethnicity, by BMI status.

Race	BMI percentiles	Difference Mean ± SD	Standardized Mean ± SD
**White**	Total (*n* = 138)	12.40 ± 14.32	1.26 ± 1.46
	<95^th%^ (*n* = 93)	9.24 ± 12.84	0.92 ± 1.27
	≥95^th%^ (*n* = 45)	18.93 ± 15.14	1.97 ± 1.57
**Black or** **African**	Total (*n* = 55)	16.09 ± 14.61	1.65 ± 1.51
	<95^th%^ (*n* = 37)	13.95 ± 14.18	1.46 ± 1.57
	≥95^th%^ (*n* = 18)	20.50 ± 13.73	2.05 ± 1.34
**Hispanic or** **Latino**	Total (*n* = 41)	18.90 ± 15.29	1.92 ± 1.57
	<95^th%^ (*n* = 19)	13.47 ± 14.19	1.38 ± 1.47
	≥95^th%^ (*n* = 22)	23.59 ± 14.95	2.38 ± 1.55
**Others**	Total (*n* = 62)	14.49 ± 14.65	1.52 ( ± 1.52
	<95^th%^ (*n* = 30)	13.77 ± 13.20	1.41 ± 1.39
	≥95^th%^ (*n* = 32)	15.75 ± 15.78	1.62 ± 1.66
**P value**	Race and Ethnicity effect	0.264	0.304
	BMI’s effect	<0.0001	<0.0001

The effect of high BMI on standardized BA advancement was found to be statistically significant (beta = 0.018, *P* < 0.0001), after adjustment for sex, age, or ethnicity. In addition, we found that male sex has a statistically significant effect on standardized BA advancement (beta = 0.834, *P* < 0.0001), as does Hispanic or Latino ethnicity vs White (beta = 0.559, *P* = 0.023, see Table 5).

**Table 5:** Multivariate analysis – Association between all explanatory variables (age, biological sex, BMI, race, and ethnicity) and standardized bone age advancement (standardized difference between bone age and chronological age).

		Model I		Model II		Model III		Model IV	
		Estimate (SE)	*P* value	Estimate (SE)	*P* value	Estimate (SE)	*P* value	Estimate (SE)	P value
**BMI**		0.019 (0.003)	<0.0001	0.020 (0.003)	<0.0001	0.019 (0.003)	<0.0001	0.018 (0.003)	<0.0001
**Age**				0.010 (0.003)	0.006	0.005 (0.004)	0.175	0.006 (0.003)	0.127
**Male vs. Female**						0.865 (0.187)	<0.0001	0.834 (0.187)	<0.0001
**Race and Ethnicity**	**Black or African vs White**							0.285 (0.220)	0.196
	**Hispanic or Latino vs White**							0.559 (0.246)	0.023
	**Others vs White**							0.200 (0.211)	0.345

Our cohort of patients for whom both the APH and the NFH were available was small, and included only 60 individual patients. We were therefore unable to perform meaningful statistical analyses to examine the combined effects of BMI, sex, race, and ethnicity on BA advancement. Table 6 provides descriptive statistics to show the differences between NFH and APH stratified by these different variables. Of the 60 patients available for this analysis, 78.3% were females, nearly half had a healthy BMI, 23% had an overweight and nearly a third had obesity. Over- or underestimation of APH was not dependent on BMI percentiles, race, or ethnicity, given the lack of directionality in APH-NFH. However, a greater proportion of males achieved NFH that was lower than the APH (chi square = 4.421, *p* = 0.036).

**Table 6:** Descriptive statistics: Near Final Height vs. Adult Predicted Height measured in cm in 60 individual patients according to variables of interests.

Characteristics	Mean (SD) or *N* (%)
***N***	60
**Biological sex (***n***, %)**	Female 47 (78.3%) Male 13 (21.7%)
**NFH vs. APH**	
NFH < APH	22 (36.7%)
Female (*n* = 14, 29.8% within sex)	
Male (*n* = 8, 61.5% within sex)	
NFH > APH	38 (63.3%)
Female (*n* = 33, 70.2% within sex)	
Male (*n* = 5, 38.5% within sex)	
**Mean NFH-APH (cm) by BMI percentiles**	
5 ≤ BMI < 85^th%^ (*n* = 28, 46.7%)	−2.2 ± 7.56
85 ≤ BMI < 95^th%^ (*n* = 11, 23.3%)	0.2 ± 6.65
BMI ≥ 95^th%^ (*n* = 19, 31.7%)	−0.62 ± 8.52
**Mean NFH-APH (cm) by BMI percentiles and sex**	
**5** **≤** **BMI < 85th**^**%**^	
Female (*n* = 23)	−0.94 ± 6.65
Male (*n* = 5)	−7.93 ± 9.59
**85** **≤ BMI** **<** **95th**^**%**^	
Female (*n* = 11)	0.6 ± 5.78
Male (*n* = 3)	−1.23 ± 10.8
**BMI ≥** **95**^**th%**^	
Female (*n* = 13)	3.56 ± 5.6
Male (*n* = 6)	−9.67 ± 6.48
**Mean NFH-APH by race and ethnicity (***n***, %)**	
White (*n* = 18, 30.0%)	0.27 ± 5.1
Black or African-American (*n* = 12, 20.0%)	−2.67 ± 7.78
Hispanic or Latino (*n* = 14, 13.9%)	0.13 ± 7.53
Other (*n* = 16, 26.7%)	1.51 ± 5.6

*BMI* Body mass index, *NFH* Near final height, *APH* Adult predicted height.

## Discussion

In this study, we found that BMI status, sex, race, and ethnicity significantly influenced BA advancement in children with isolated, idiopathic PA. Specifically, higher BMI and male sex were significantly associated, and Hispanic or Latino ethnicities showed a trend towards more advanced BA.

Determination of skeletal maturity constitutes an integral component of the diagnostic endocrine workup in suspected early puberty. Current worldwide practice is to use the Greulich and Pyle atlas,^11,17^, which was established in the 1930s using hand and wrist radiographs of 1000 Caucasian children from a medium-high socioeconomic status in North America. This atlas also provides standards for the percentage of growth achieved at each BA and forms the basis for final height prediction using the Bayley-Pinneau tables.^14^ Significant BA advancement (more than two SDs compared with the chronological age) would usually prompt further laboratory evaluation of underlying pathophysiology and could potentially affect decisions concerning treatment initiation to delay puberty and preserve adult height (e.g., GnRH analogs). Our findings regarding differences in bone age advancement between children of different racial and ethnic backgrounds have been reported before in a large cohort of children with normal skeletal development (BA within 2 SDs according to the Brush Foundation tables).^18^ Our study confirms the findings concerning advanced BA in Hispanic children and adds to the literature by examining the combined effects of ethnicity, BMI status, and biological sex on BA advancement and specifically in children evaluated for PA. These cross-racial differences may reflect substantial flaws in continuing to use bone age standards derived from a relatively homogeneous white population for patients from diverse ethnic and racial backgrounds, particularly when these standards are potentially used to determine whether further evaluation for pathophysiology is pursued. It is also important to recognize that race and ethnicity are complex constructs that should not be interpreted as direct biological determinants of health outcomes. Therefore, caution is advised when extrapolating these findings to avoid perpetuating biases or disparities in healthcare. Further research is needed to understand the underlying factors contributing to differential bone age advancement, and to ensure that evaluation and treatment approaches are equitable for individuals from all racial and ethnic backgrounds.

Obesity is a common finding in PA, and both PA and childhood obesity are independently associated with BA advancement,^6,7,19,20^ increased DHEAS levels^19,21^, as well as an earlier acceleration in growth velocity.^20,22,23^ The mechanisms underlying BA advancement in these two conditions could be related to hyperandrogenemia,^7^ increased aromatization of androgens to estradiol in adipose tissue, and lower sex hormone binding globulin levels in the setting of obesity,^24^ all eventually leading to increased estradiol concentration at the growth plate, thus facilitating bone maturation and epiphyseal fusion. Of note, the enzyme aromatase, which catalyzes the conversion of androgen precursors to estrogen, is specifically expressed in the human growth plate,^25^ and leptin, which is increased in the setting of obesity, stimulates the activity of aromatase.^26^ Consistent with the literature, in our study, most children with isolated PA presented with some degree of BA advancement regardless of their BMI status, and 61% of children had either overweight or obesity. Additionally, when comparing the degree of BA advancement according to BMI stratification, we found that in each BA advancement category (any advancement, advancement greater than 1 SD, and greater than 2 SDs), the proportion of children with obesity was significantly greater. Among those with BA advancement greater than 2 SDs, less than a fifth of children presented with healthy BMI (5–85^th%^) while almost half had obesity.

When examining differences in BA advancement based on biological sex, we found that boys with isolated PA demonstrated greater advancement in BA even when accounting for BMI. To the best of our knowledge, this is the largest study to date evaluating sex differences in BA advancement in isolated PA. Most previous studies examining BA advancement in PA included only girls^6,9,10^ or did not report sex differences in their small mixed cohorts.^7,8,27^ Santos-Silva et al., who examined 82 Portuguese Caucasian children (15% boys), did not find sex differences in anthropometrics, androgen levels, or BA advancement.^28^ Conversely, in a medium-sized, multi-ethnicity cohort (57% White, 17% Hispanic, 11% Black), BA advancement and DHEAS levels were significantly greater in boys, while BMI did not differ between the two groups.^29^ Premature adrenarche is diagnosed 5-fold more commonly in girls than in boys.^4^ In a large survey Finnish study^30^ of pre-pubertal children (age range 6.8-8.9 years), DHEAS levels did not differ between boys and girls. However, clinical signs of adrenarche were more common, while biochemical evidence for adrenarche without clinical manifestation was less prevalent in girls than in boys. Among the children with early biochemical adrenarche (DHEAS levels > 37 mcg/dL) and clinical signs of premature adrenarche, 8.6% were girls, and only 1.8% were boys. Similarly, among those with early biochemical adrenarche and without any clinical signs, 8% were girls and 17% were boys. Theoretically, it is possible that in boys with PA a greater exposure to adrenal androgens is needed to result in clinical signs, such that greater BA advancement is observed when they present to medical evaluation. Further studies are required to investigate the biological mechanisms underlying sexual dimorphism in premature adrenarche-related BA advancement.

Whether BA advancement in isolated PA affects final adult height relative to the MPH, or affects the applicability of height prediction, is currently uncertain. Three prospective case-control studies from Spain,^10^ Finland^9^ and Israel^6^ found that girls with PA had adult height which was comparable with their MPH. Importantly, girls in these studies had a mean BMI in the healthy range and were reported to be Caucasian in two of the studies, while in the third study, race and ethnicity were not reported. In another study from the US with an estimated proportion of 55% Black patients, adult predicted height was only slightly lower than the MPH in girls with BA advancement greater than 2 SDs (160.8 vs. 163.7 cm, *p* = 0.04), but it did not differ in boys or girls with lesser BA advancement.^8^ In contrast to these studies, in children originating from South and central America (64% Hispanic), the majority of whom had overweight or obesity, half had adult predicted height, which was at least 1 inch below the MPH, and 20% of the study population had a height difference of more than 3 inches below the target height. 72% of the latter group demonstrated BA advancement greater than 2 SDs.^31^ In summary, the synthesis of previous studies suggests that the effects of BA advancement on final and predicted adult heights are related to BMI status, sex, and ethnicity. While the subset of patients with NFH measurements was too small to draw meaningful statistical conclusions, we observed a trend toward slightly lower NFH compared to APH in males. However, given the limited sample size and wide standard deviations, this difference should be interpreted with caution. Further, in our cohort we found that being Hispanic or Latino vs. White had a significant effect on BA advancement, emphasizing the need to conduct well-designed studies to assess bone age and height at various ages in a racially and ethnically diverse cohort of children and assess whether the mathematical relationship between bone age and final height determined in the Greulich and Pyle cohort remains accurate in a more diverse group.

Our study has certain limitations; the first two relate to the retrospective nature of this study. First, NFH, calculated as the final height available in girls older than 14 years and boys older than 16 years of age, was available for only 60 individual participants. Only 13 boys were included in this sample, with significant inter-subject data variability reflected by large standard deviations, restricting our ability to conduct robust statistical analysis. We were therefore unable to examine the effects of ethnicity, sex, and BMI on the accuracy of predicted adult height compared to final height in this relatively small sample. Second, we did not include a control group of children without clinical signs of PA. Additionally, we employed formal radiologist readings to determine the BA, which might have been influenced by the patient’s name, sex, and age visible in the electronic medical record. While the substantial size of our cohort study helped to mitigate some of the variability and potential bias in BA interpretation, future research could consider analyzing the BAs by combining input from two individual blinded interpreters and/or by a dedicated computer software to further enhance the robustness of our findings. Because laboratory results were not consistently available in the medical records, we were unable to determine the proportion of patients who underwent biochemical testing. This limits our ability to fully characterize the diagnostic workup and to confirm idiopathic PA in all cases. As a result, it is possible that a small number of patients included in the analysis had an alternative etiology for their presentation, which may have introduced some degree of misclassification bias. Finally, we did not include data concerning birth weight or laboratory evaluation in this study. As mentioned above, previous studies have consistently demonstrated the association of being born small for gestation age and/or having increased DHEAS levels with BA advancement, BMI status and sex. However, to the best of our knowledge, the relationship of DHEAS levels to BA advancement in children from different races and ethnicities has not been methodically examined before. Since variations in androgen levels may exist by race and ethnicity,^32^ they should be studied in the context of PA. We also acknowledge the complexity of race and ethnicity as a variable, as it may reflect relevant underlying genetic differences, but may also reflect epigenetic and physiological effects of different lived experiences, including resource scarcity and discrimination. We report the differences in bone age advancement based on race and ethnicity because this information raises questions regarding the appropriateness of our current bone age standards and may reflect important physiological processes. We do *not* advocate for using race and ethnicity in clinical algorithms to determine further evaluation and management, as this may further perpetuate existing disparities. While our findings highlight an association between elevated BMI and advanced bone age in patients with premature adrenarche, we do not suggest that bone age assessment should be routinely performed in all patients with this condition. Rather, decisions regarding the use of bone age testing should be made on an individual basis, depending on factors such as growth trajectories, timing and progression of pubertal signs, and provider judgment.

In reviewing skeletal maturation in this relatively large, racially and ethnically diverse population of boys and girls with PA, we show that BA advancement is highly dependent on BMI status, sex, and ethnicity. Further studies are needed to fully understand and disentangle the effects of these parameters on height prediction and adult height. Also, given the fact that children with premature adrenarche are at increased risk for metabolic complications, it is important to fully understand the effects of sex and adiposity on the pathophysiology of PA in these children.^33,34^

## Data Availability

The datasets generated during and/or analyzed during the current study are available from the corresponding author on reasonable request.
